# Visible-Light-Driven Photoactivity of Copper/PVA Composite Films against Murine Coronavirus

**DOI:** 10.1021/acsomega.5c02542

**Published:** 2025-10-01

**Authors:** Aline L. Schio, Michele S. de Lima, Marina D. Giustina, Rafael D. Cadamuro, Catielen P. Pavi, Alexandre F. Michels, Gislaine Fongaro, Mariana Roesch-Ely, Carlos A. Figueroa

**Affiliations:** † Postgraduate Program in Materials Science and Engineering, University of Caxias do Sul, Caxias do Sul, Rio Grande do Sul 95070-560, Brazil; ‡ Biotechnology Institute, University of Caxias do Sul, Caxias do Sul, Rio Grande do Sul 95070-560, Brazil; § Laboratory of Applied Virology, Department of Microbiology, Immunology, and Parasitology, Federal University of Santa Catarina, Florianópolis, Santa Catarina 88040-900, Brazil

## Abstract

In light of the Severe Acute Respiratory Syndrome coronavirus 2 (SARS-CoV-2) pandemic in 2019, the global scientific community has focused their research on developing virucidal materials to mitigate the spread of viruses including self-disinfecting surfaces capable of inactivating viruses that are in contact with the material. Copper stands out in this context due to its intrinsic ability to disrupt viral structures and inhibit replication mechanisms, making cooper-based materials a powerful tool. Furthermore, copper absorbs light in the visible region, a property that has not been fully studied or reported in the literature considering its photoactivity on viruses. To fill this gap, in the present study, composite films containing 2% w/v of copper micro- and nanoparticles and copper oxide (I) microparticles in poly­(vinyl alcohol) (PVA) were synthesized by drop-casting. The films were evaluated against enveloped murine hepatitis virus type 3 (MHV-3) under dark and white light illumination. Using the L929 mouse fibroblast cell line, only the film with copper microparticles showed no cytopathic effect under illumination, indicating virucidal photoactivity. By performing the integrated cell culture and reverse transcription quantitative PCR (ICC-RT-qPCR) assay, it was found that the film promoted a 43.1% reduction of viral load when illuminated, while in the dark, the reduction was lower than 7%. Thus, it was possible to obtain photoactive composite films with relatively low-cost copper microparticles by a conventional deposition technique. These promising results contribute to advancing research on visible-light-driven materials and photofunctional surfaces that use solar energy as a power source, offering innovative solutions to combat the spread of pathogens.

## Graphical abstract

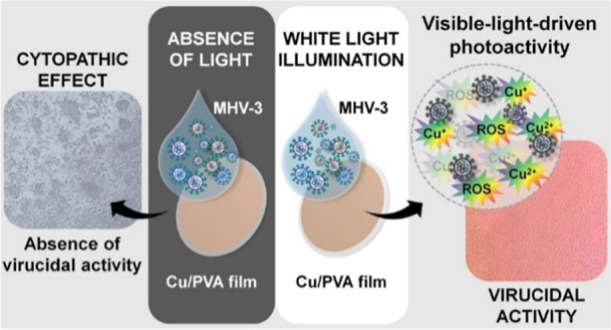

## Introduction

Since the first pandemic caused by a coronavirus, designated as SARS-CoV-2, in late 2019, more than 7 million deaths have been reported worldwide due to COVID-19. During the outbreak, one of the measures implemented worldwide to mitigate the spread of the virus was the use of face masks. This measure was maintained even after the start of vaccination, as it takes months to administer the doses required to achieve the proposed efficacy rate.^1–3^

Additional measures to control the spread of the virus included basic sanitation and the use of chemical agents, primarily aimed at the disinfection of inanimate objects and surfaces. Ozone and ultraviolet radiation have also demonstrated virucidal efficacy along with natural compounds such as essential oils and chitosan. However, despite the wide range of options, none of these agents provide lasting decontamination, as repeated exposure to viral loads results in recontamination.^4–7^

This scenario, combined with studies demonstrating that SARS-CoV-2 and several other viruses can survive for hours or even days on inanimate surfaces and objects, underscores their significant role in the spread of pathogens. This has highlighted the importance of researching and developing materials and coatings with intrinsic virucidal activity.^4,5,8,9^

Among the most studied materials in this field, copper stands out. Copper has been known as a disinfecting agent since ancient times. According to Grass, Rensing, and Solioz, the Smith Papyrus, an Egyptian text that dates back to 2200 B.C., mentions the use of copper to treat wounds and contaminated water.^10^ As the centuries passed, its use kept growing along, and in 2008, it was the first metal agent recognized by the United States Environmental Protection Agency (EPA) to destroy pathogenic microorganisms.^11^ During the pandemic of 2019, studies demonstrated that copper surfaces could eliminate viable SARS-CoV-2 within just 4 h, while in comparison, the virus remained viable for up to 24 h on cardboard and up to 48 h on plastic and stainless steel.^12^

However, despite this long-standing knowledge of copper’s properties, its biocidal and virucidal photoactivity remains largely underexplored. This research gap is even more pronounced, concerning the use of metallic copper particles. Most studies have focused on copper’s photoactivity when combined with semiconductors,^13–15^ carbon-based materials,^16,17^ and so on.^18–20^ Notably, no studies have evaluated the photocatalytic effects of copper microparticles against viruses. Since the advent of nanotechnology, research efforts have predominantly shifted toward investigating material properties at the nanoscale, often seen as a benchmark of innovation.

Therefore, based on our previous findings that visible light enhances the bactericidal activity of copper particles^21^ and that incorporating them into a polymer matrix preserves their activity,^22^ the present work evaluated the virucidal photoactivity of metallic copper micro- and nanoparticles and copper oxide (I) microparticles, incorporated into poly­(vinyl alcohol) (PVA), in the presence and absence of white light illumination. PVA was used as a polymeric matrix due to its noncytotoxicity, biocompatibility, and film-formation ability. Also, as hydrophilic, it can release the particles incorporated to provide a constant interaction with the medium.^23–25^ The tests were performed with murine coronavirus type 3, also known as murine hepatitis virus type 3 (MHV-3).

## Experimental Section

### Materials

Copper microparticles (CuMPs) were purchased from Metal Pó (code D2200065), while copper nanoparticles (CuNPs, code 774081), copper oxide (I) microparticles (Cu_2_OMPs, code 208825), and poly­(vinyl alcohol) (PVA, code 363146) were purchased from Merck. A micrograph of each particle sample obtained by scanning electron microscopy (SEM; Shimadzu SSX-550 Superscan) is presented in Figure 1a–c, followed by the respective particle size distribution histogram below estimated using the ImageJ software^26^ (Figure 1d–f). The full characterization of the particles is reported in our previous work, including the optical absorbance in the visible range (UV–vis, Shimadzu Spectrophotometer, model UV-2600i), the band gap calculated by the Tauc plot using the Kubelka–Munk function, the crystalline structure, phase, and crystallite size determined by X-ray powder diffraction (XRD; Shimadzu LabX XRD-6000 with Cu Kα radiation, λ = 1.5406 Å), and the surface chemical composition analyzed by ex situ X-ray photoelectron spectroscopy (XPS; Thermo Alpha 110 Hemispherical Analyzer with a 10 kV Al Kα nonmonochromatized radiation source).

**Figure 1:**
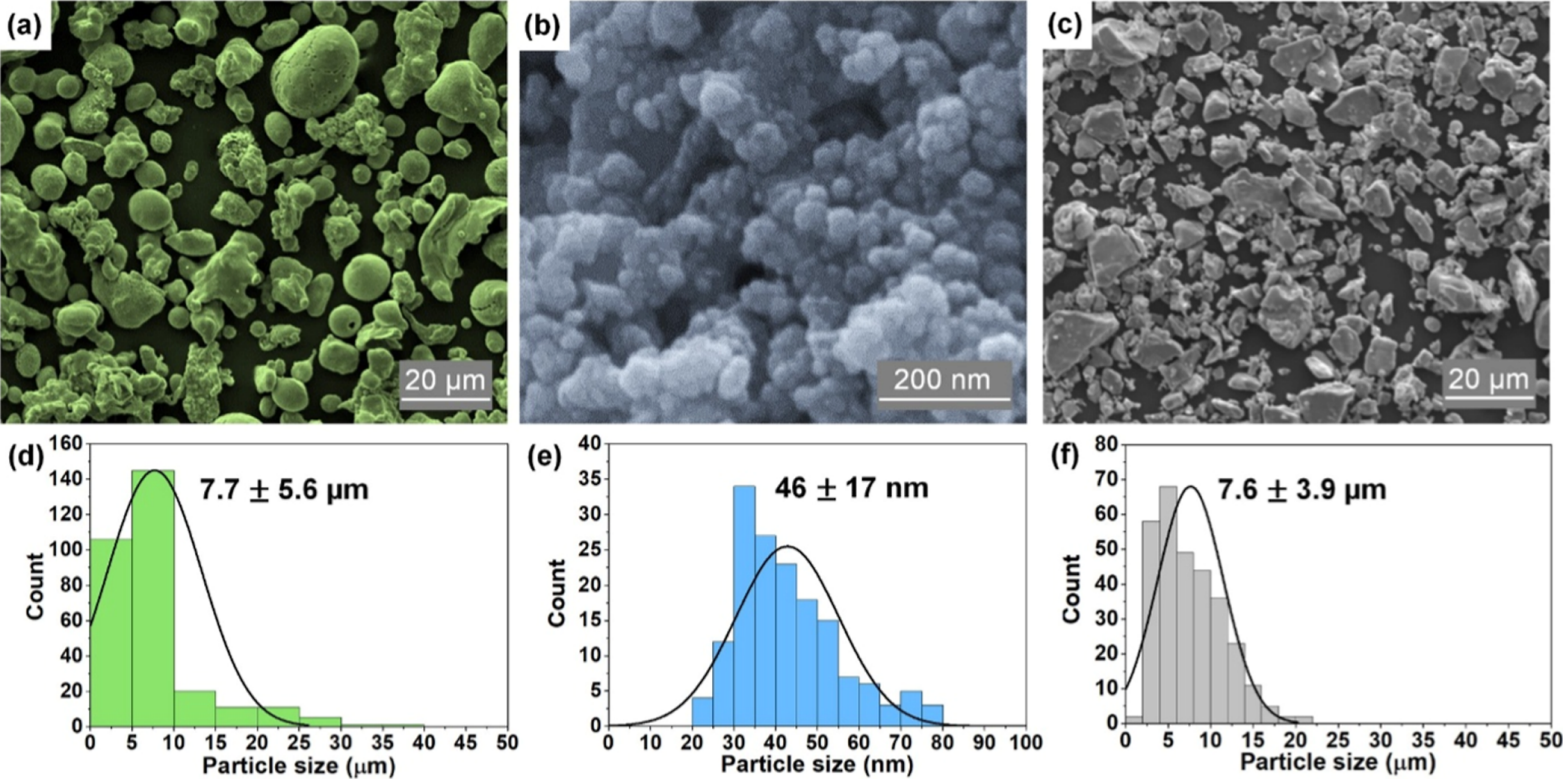
Micrographs of CuMPs (a), CuNPs (b), and Cu_2_OMPs (c) obtained by SEM and their respective size distribution histograms below presenting the mean ± SD of measurements (d–f).

This previous work also presented the bactericidal photoactivity of the particles in suspension against *Staphylococcus aureus* and *Escherichia coli* in the absence of light and under illumination. Our results showed that the bactericidal activity under illumination provided a percentage increase in log reduction values of 65.2% for *S. aureus* and 166.7% for *E. coli* when compared to the assays in the absence of light. Summarizing, it demonstrated the visible-light-driven photoactivity of copper particle suspensions combating bacteria with size- and dose-dependent activity.^21^

### Composite Film Synthesis and Characterization

Copper/PVA composite films, as well as the pure PVA film, were obtained by drop-casting following the same methodology described in previous work.^22^ The PVA matrix solution was prepared following methods reported in the literature.^24,27^ PVA 5% w/v suspension was heated at 80 °C under constant stirring for 4 h. The solution was drop-cast over 13 mm diameter glass slides and dried at 70 °C for 1 h to produce a pure PVA film. In a separate set of PVA solutions, copper particles were individually added at a concentration of 2% w/v. The composite suspensions were subjected to magnetic stirring combined with ultrasonic probe treatment for 10 min before being drop-cast over the glass slides and then dried at 70 °C for 1 h.^17,24,27^

The distribution of the particles into the PVA was analyzed by scanning electron microscopy (SEM; Shimadzu SSX-550 Superscan) and the depth composition by glow discharge optical emission spectroscopy (GDOES; Horiba Scientific, GD-Profiler 2). The film roughness was analyzed by atomic force microscopy (AFM, Shimadzu, SPM-9700). The optical absorbance in the visible range of the films was obtained in a Shimadzu Spectrophotometer (UV-2600i), and the band gap was estimated. The films were also subjected to wettability analysis by contact angle measurement (Phoenix, P150).

Copper particles are observed at the surfaces of the composite films (Figure 2a–c), especially in the CuNPs/PVA film, where a higher amount of nanoparticles can be observed since they have a significantly higher surface-to-volume ratio when compared to microparticles, which makes them less susceptible to sedimentation.^28,29^ Figure 2d presents some of the main characteristics of the composite films and the pure PVA one. As for the thickness, according to SEM and GDOES analysis, the films were found to have an uneven thickness, which is also attributed to the deposition technique used. AFM results showed that the incorporation of the particles increased the surface roughness to 67%. The band gap value, which has a direct influence on photocatalytic applications, was obtained by the Tauc plot technique and found to be between 2.0 and 2.2 eV, all in the visible light spectrum as desired.^30,31^ As for the hydrophilicity, the particle incorporation into the PVA matrix promoted a percentage increase of about 50% in the contact angle; however, the composite films remained hydrophilic, which is interesting for the proposed application.^32,33^ More details and characterization results of the composite films, including XRD, FTIR, and TGA analysis, are reported in our previous work, including their bactericidal photoactivity. Within 24 h, all composite films showed above a 6 log reduction in bacterial load (>99.9999% removal), making them promising candidates for testing against viruses.^22^

**Figure 2:**
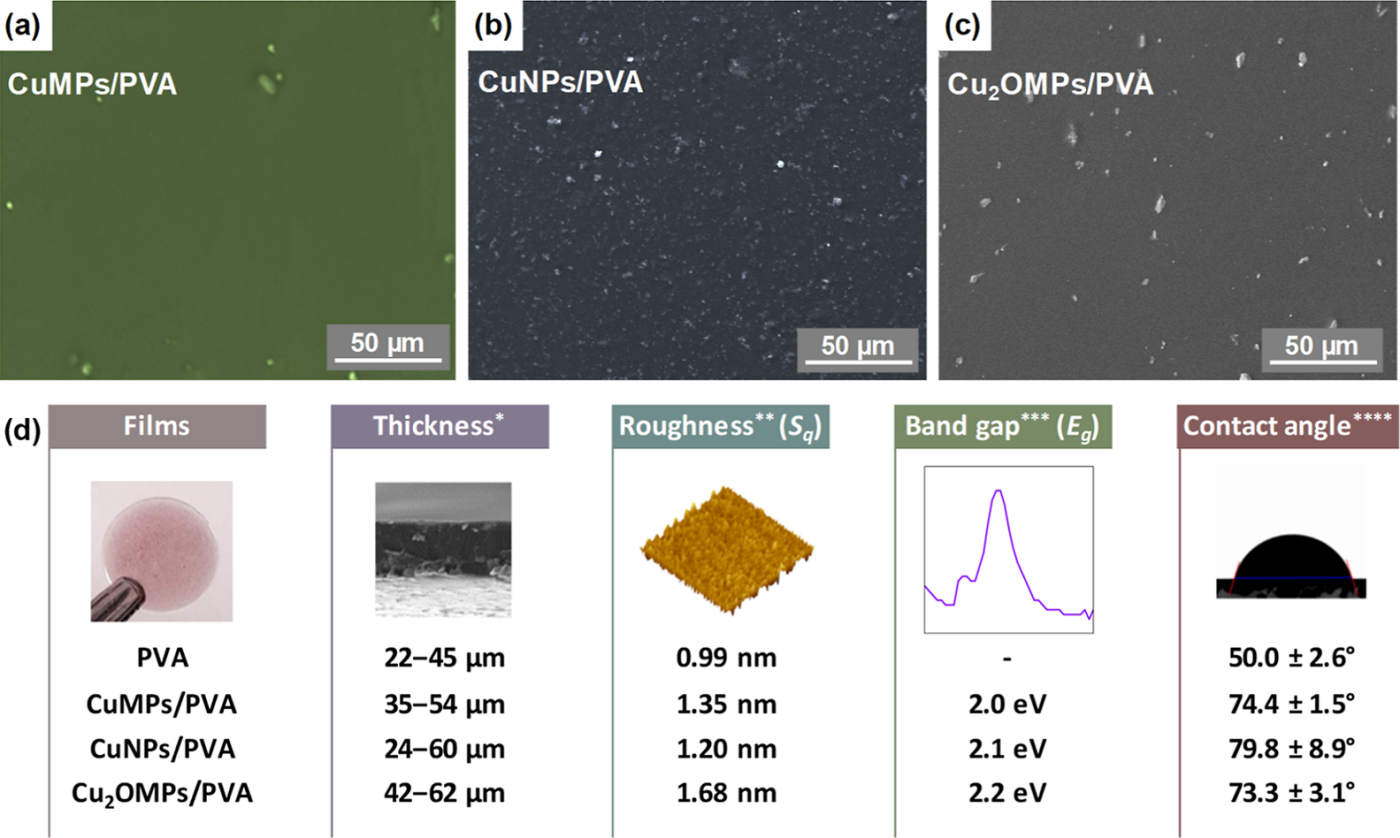
SEM micrograph of the PVA films containing 2% w/v of CuMPs (a), CuNPs (b), and Cu_2_OMPs (c) and some of their characteristics (d). *Thickness is presented as a range (minimum–maximum) based on multiple measurements from SEM micrographs, in agreement with GDOES. **Roughness is expressed as root mean square roughness (*S*_*q*_) of multiple measurements from AFM analysis. ***Band gap values (*E*_g_: energy gap) are reported as an estimated result from diffuse reflectance spectroscopy using the Tauc plot method. ****Contact angles are reported as the mean ± SD (standard deviation) of three measurements.

### Cell Culture and Virus Titration

The L929 cell line (ATCC CCL1), stored in a cryogenic tube in liquid nitrogen containing 90% fetal bovine serum (FBS) and 10% dimethyl sulfoxide (DMSO), was thawed at room temperature and transferred to a sterile 75 cm^2^ cell culture flask containing minimum essential medium (MEM, Gibco), supplemented with 10% fetal bovine serum (FBS, Merck). The flask was placed in an incubator at 37 ± 2 °C with an atmosphere containing 5% CO_2_.

After 48 h, cell suspensions (3.3 × 10^5^ cells mL^–1^) were prepared. The cell monolayer in the flask was washed twice with phosphate-buffered saline (PBS), followed by the addition of 500 μL of trypsin (Merck, code T4799). The flask was incubated for 5 min at 37 ± 2 °C with a 5% CO_2_ incubator, and 10 μL of the suspension was mixed with 10 μL of trypan blue (Invitrogen, code T10282) for viable cell counting using an automated Countess cell counter (Invitrogen). Subsequently, 24-well plates were prepared with a cell density of 2.5 × 10^5^ cells per well. After 24 h of incubation in the 5% CO_2_ incubator, the cell monolayers were ready for viral titration and the virucidal assay.

The MHV-3 viral stock, also stored at −80 °C, was thawed at room temperature. To determine the viral titer, the 50% tissue culture infectious dose (TCID_50_) was calculated using the Reed–Muench method.^34^

### Virucidal Photoactivity Test

The virucidal photoactivity of the films was evaluated using a neutral white LED light source from Thorlabs, Inc.^21^ The test was developed according to the representative scheme in Figure 3 based on ISO 21702:2019 with modifications.^35^ Before each test, all samples were sterilized in a chamber with UV irradiation for 2 h on each side of the surface.

**Figure 3:**
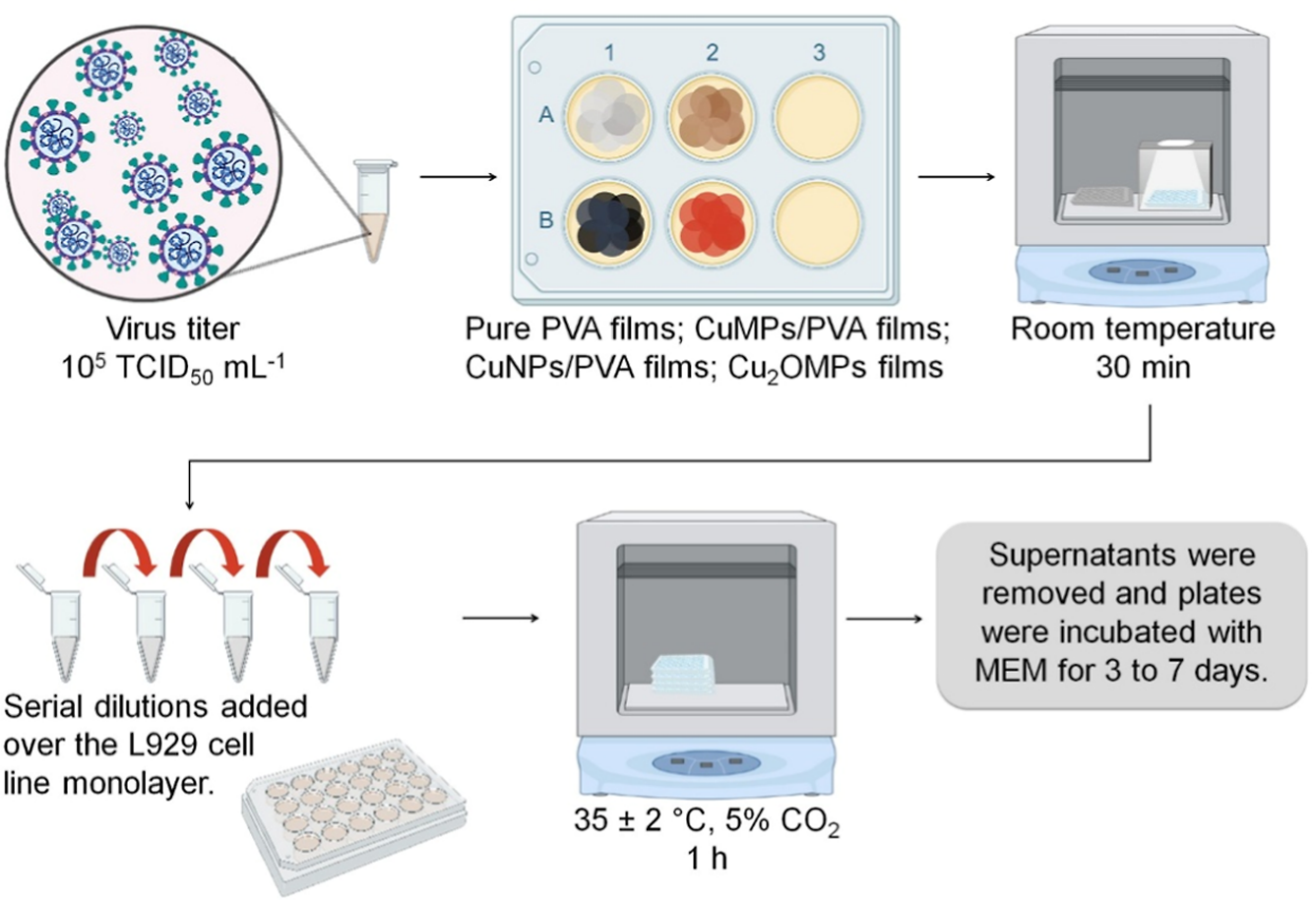
Representative scheme of the assay to evaluate the virucidal photoactivity of the films. Created with BioRender.com.

The assay consisted of direct contact between the films and the viral suspension of MHV-3 at a titer of 10^5^ TCID_50_ mL^–1^ for 30 min in the presence and absence of white light illumination at room temperature. After this period, the viral suspensions treated were eluted and diluted in 2 mL of unsupplemented MEM. From this solution (10^4^ TCID_50_ mL^–1^), a serial dilution of 1:10 was performed to obtain a second viral titer, 10^3^ TCID_50_ mL^–1^.

With these prepared solutions and the viral controls, aliquots of 100 μL were added to the 24-well plates previously prepared with the L929 cell monolayer. After 1 h in an incubator at 37 °C with 5% CO_2_, the solutions from each well were removed, and the plates were incubated once more with 750 μL of MEM supplemented with 10% FBS.

After 3 to 5 days of incubation, the plates were examined under an inverted microscope to assess the presence or absence of the cytopathic effect (CPE). From the most promising sample, integrated cell culture and reverse transcription quantitative PCR (ICC-RT-qPCR) was performed. The supernatant was removed from the treated 24-well plates, and the cell monolayers were washed three times with PBS. Then, 200 μL of trypsin was added to each well, and the plate was incubated for 2 min. After this period, 500 μL of MEM was added to each well to resuspend the cells, which were then centrifuged for 2 min at 2000 rpm. The supernatant was discarded, and the cells were resuspended in 200 μL of MEM for nucleic acid extraction. Extraction was performed using magnetic beads and following the protocol of the RNAdvance Viral Bind extraction kit (Beckman Coulter). Nucleic acid quantification of the samples was performed by using a NanoVue spectrophotometer (Avantor).

### Real-Time Quantitative PCR

Reverse transcription quantitative PCR (RT-qPCR) was performed as described by Hernroth et al. (2002)^36^ with adaptations, in a StepOne Plus Real-Time PCR System (Applied Biosystems) using the QuantiNova Probe RT PCR kit (Qiagen). Each sample was analyzed in triplicate. Ultrapure water was used as the nontemplate control for each assay. The amplification results were compared with the viral control, containing the genomic copies of the MHV-3 without the interference of the films. The cycling conditions, primers, and probe sequences are listed in Supporting Information.

### In Vitro Cytotoxicity Assay

A 100 μL portion of the murine fibroblast cell line L929 (ATCC CCL1) was added into 2 sterile 96-well plates with a seeding density of 5 × 10^4^ cells per well, which were subsequently incubated at 35 ± 2 °C in a 5% CO_2_ atmosphere for 24 h. In parallel, to two 24-well plates, 220 μL of DMEM medium (Gibco, low glucose) supplemented with 10% FBS and 1% PSA was added over the pure PVA films and the composites, and both were incubated at 35 ± 2 °C for 24 h, one in the dark and the other under visible light illumination. After the first day of testing, the medium from the 96-well plates was aspirated, and 100 μL of the solution of the medium treated with the polymeric films was added. These plates were, once again, incubated with 5% CO_2_ (both in the dark), and after 24 h, the medium was aspirated to add 100 μL of the MTT solution (3-(4,5-dimethyl-2-thiazolyl)-2,5-diphenyl-2*H*-tetrazolium bromide) diluted in DMEM medium in a 2:3 (v/v) ratio. The treated cells remained in contact with the MTT for 2 h at 35 ± 2 °C with CO_2_, and finally, the solution was aspirated, and 100 μL of dimethyl sulfoxide (DMSO) was added so that, after 20 min of shaking, the absorbance of the solutions could be read (Figure 4). Absorption values were measured at 570 nm using a microplate reader (Multiskan FC, Thermo Fischer). Cell viability represents the ratio between viable treated cells (sample) and control (cells not treated with the films).^6,37–39^

**Figure 4:**
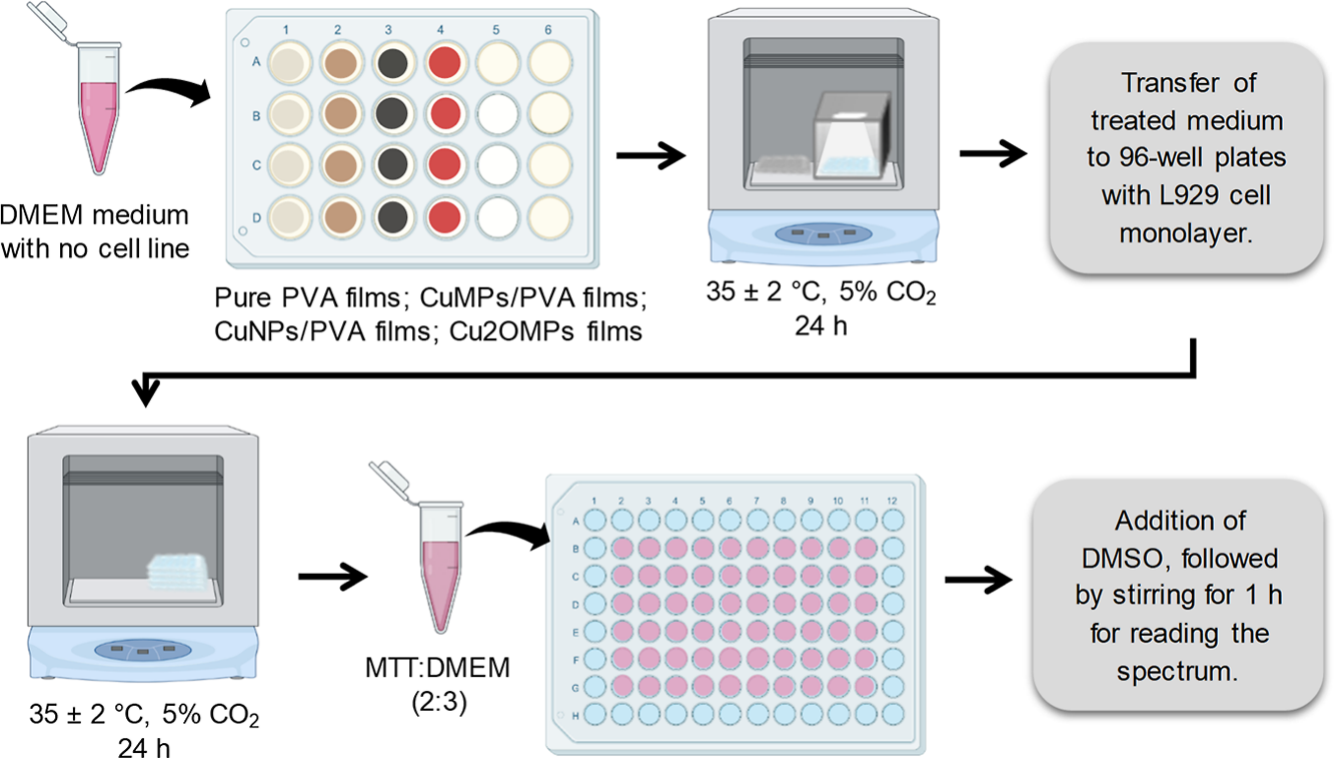
Representative scheme of the assay to evaluate the cytotoxicity of the films. Created with BioRender.com.

## Results and Discussion

### Virucidal Photoactivity

At the end of the last incubation of the 24-well plates from the virucidal photoactivity test of the composite films, all of the wells were examined under an inverted microscope. The cytopathic effect results are expressed in Table 1 as a function of the MHV-3 virus’s cytopathic effect on the L929 strain according to each treatment performed. This last experiment made it possible to filter the most promising composite film to be tested in a more sensitive, precise, and quantitative analysis, the ICC-RT-qPCR. The CuMPs/PVA film was the only one to present relative potential virucidal activity, exclusively under illumination, where no cytopathic effect was observed.

**Table 1:** Cytopathic Effect of the MHV-3 Virus on the L929 Cell Line under Different Treatments^a^

treatment	absence of light	illumination with white light
cell control	–	–
virus control	+	+
PVA	+	+
CuMPs/PVA	+	–
CuNPs/PVA	+	+
Cu_2_OMPs/PVA	+	+

a +: Presence of cytopathic effect; −: absence of cytopathic effect.

Thus, the ICC-RT-qPCR experiment was performed in triplicate to evaluate, more specifically, the virucidal activity of the CuMPs/PVA film under both illumination conditions. Based on the average of three measurements of viral particles, the composite film presented a 43.1% reduction in viral load when exposed to white light, as compared to the viral control (mean of 1.7 × 10^11^ viral particles), while the reduction in the dark was only 6.8% (Figure 5).

**Figure 5:**
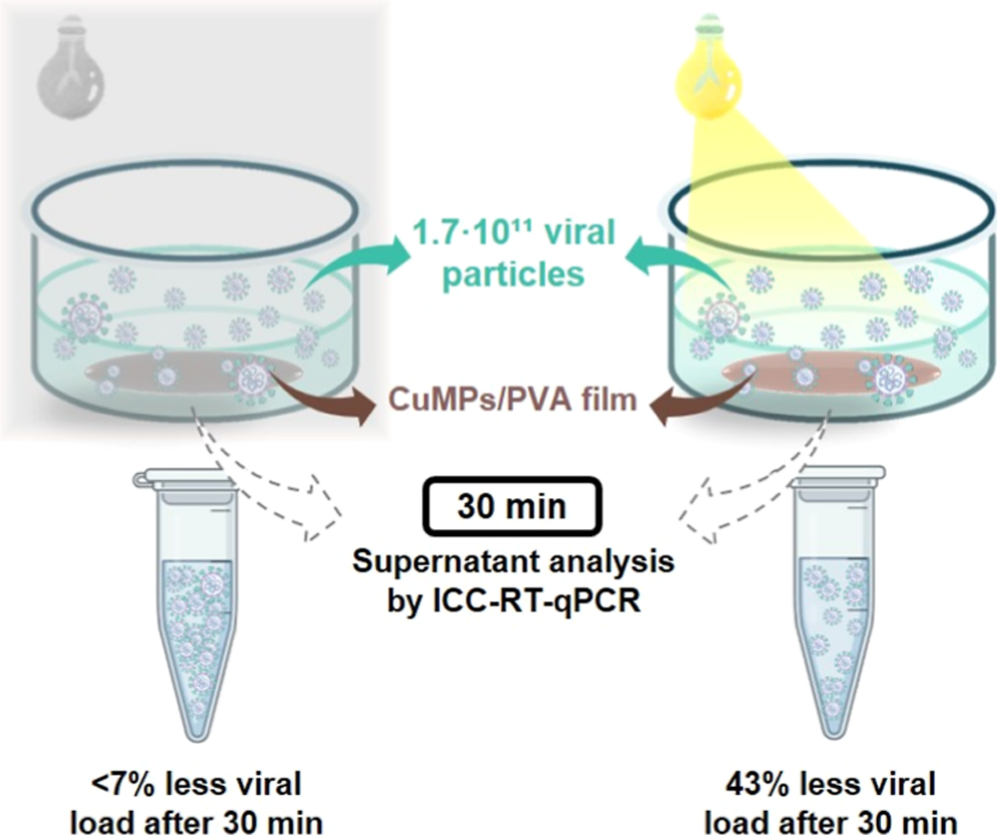
Schematic representation of the ICC-RT-qPCR result: viral load reduction after exposure to CuMPs/PVA films under different illumination conditions. Created with BioRender.com.

According to different specific studies in this area, the main mechanism of the virucidal (preinfection) activity of copper is disinfection by contact. The adhesion of copper particles, including Cu^+^ and Cu^2+^ ions and generated reactive oxygen species (ROS), to the viral wall breaks the protection and/or the capsid proteins, denaturing the genome and destroying the structure and function of proteins and nucleic acids.^27,40–42^

The higher virucidal activity of the composite film exposed to visible light may be attributed to enhanced ROS generation via photocatalysis. Commonly associated with the use of a semiconductor as a photocatalyst, photocatalysis should be understood as a process that combines photochemistry with heterogeneous catalysis. There are four main steps in this process: (i) the absorption of light irradiation at appropriate wavelengths (with sufficient energy), (ii) the excitation of electrons from the valence band to the conduction band of the semiconductor, (iii) the migration of the generated electron–hole (e^–^/h^+^) pairs to the surface of the semiconductor, and (iv) the redox reactions that result in the formation of ROS. In the presence of viruses, ROS induce oxidative damage to capsid proteins, disrupting the structure and function of both proteins and nucleic acids, thereby preventing infection.^43–46^ Regarding metals, defined as materials in which the conduction band is partially filled, only energy levels above a specific energy called the Fermi level can accept excited electrons. Consequently, all incident radiation can be absorbed regardless of its wavelength, also promoting a redox environment that leads to a biocidal effect.^30,45^

Therefore, the combined effect of metallic copper and its surface oxides^21^ contributes to the notably higher photoactivity observed. Figure 6 presents a schematic representation of the CuMPs/PVA film under dark and visible light conditions, along with photographs of treated cell monolayers. A cytopathic effect is characterized by cellular lesions and morphological changes due to viral infection (left microscopy), while its absence indicates virucidal activity of the tested material, evidenced by a uniform and well-distributed cell monolayer (right microscopy).^47–49^

**Figure 6:**
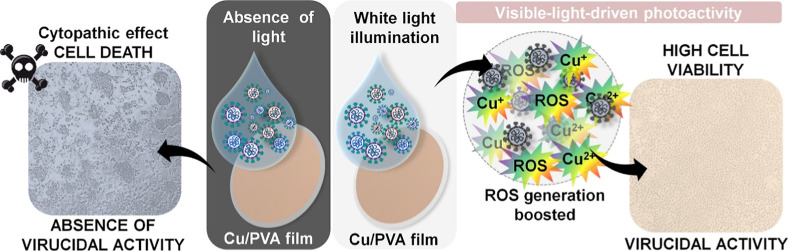
Schematic representation of the CuMPs/PVA activity in the absence and presence of white light illumination against MHV-3. Created with BioRender.com.

Regarding the numeric result, although the virucidal activity remained below satisfactory levels for biological applications (defined as a reduction of at least 90%), the results indicate the influence of light in enhancing the virucidal action of copper microparticle-based films. Consequently, new experiments regarding the optimization of experimental parameters, such as irradiation intensity and exposure time, can be adapted to improve the virucidal photoactivity up to satisfactory levels. Despite this, considering both a low-cost deposition technique and a virucidal agent (microparticles), this study contributes to an important research field with significant market potential.

In addition, it is important to recognize the dose-dependent response, since several articles have already shown that, among different surface materials, the fastest decomposition of SARS-CoV-2 was observed on copper-based surfaces.^12,50^ In Kubo et al.’s work,^51^ CuSO_4_ nanoparticles were incorporated into cellulose fibers (7.5%) by electrospinning for the production of facial masks, and it reduced influenza A virus titers by 1.1–1.8 log after minutes of exposure and by 1.6–1.8 log after 1 h. For SARS-CoV-2, the reduction was 0.38 log after 5 min of exposure and reached total inactivation within 1 h. Díaz-Puertas et al.^37^ incorporated ceramic-coated silver nanoparticles into thermoplastic polyurethane (TPU) plates to combat a fish pathogen and reported that the activity of the composite (which achieved a 75% reduction in viral load) was dependent not only on the contact time but also on the test temperature.

### Cytotoxicity of the Films

Within 24 h, no cytotoxicity of PVA was observed, as the average cell viability remained above 100%, indicating cell growth (replication) compared to the control and, consequently, biocompatibility. As for the composite films, all exhibited high relative cytotoxicity, since the average cell viability ranged between 13% and 16% (Figure 7). The recommended threshold to ensure biosafety is above 70%.^38^ Thus, we adapted the assay to be conducted with a 30 min incubation period, replicating the contact time used in virucidal tests. In this shorter period, we observed that light influences the cytotoxicity of the PVA-CuMPs film: in the absence of light, the average cell viability was 92%, while under illumination, it decreased to 67%. This result is justified by the material’s photoactivity, already demonstrated in previous assays. Regarding the film containing CuNPs, cytotoxicity was evident even within this short time frame, confirming the strong oxidative power of the nanoparticles. Finally, the PVA-Cu_2_OMPs film showed higher cell viability values, but still within the range considered cytotoxic.

**Figure 7:**
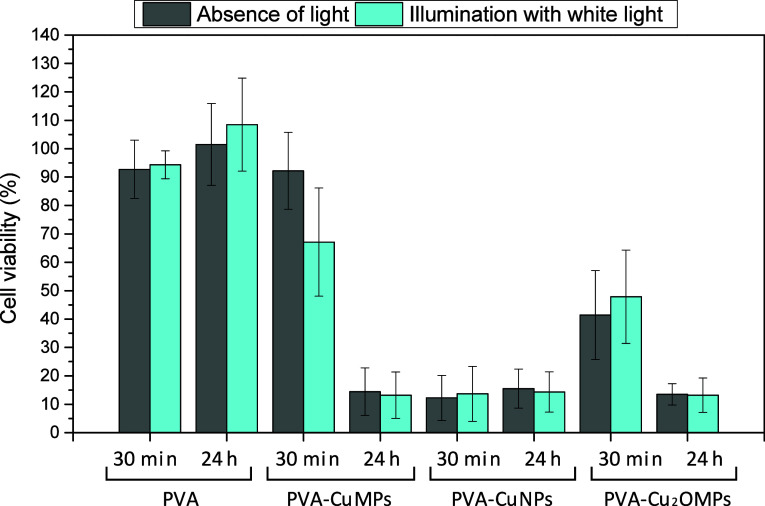
Cell viability of the L929 cell line by the MTT assay using PVA films and composite films. Error bars represent ± SD, and data are from multiple experiments.

Copper acts as a cofactor in several redox enzymes, being essential for the functional activity of oxidoreductases and for proteins involved in the intracellular transport of electrons and oxygen. This fundamental role helps explain why copper has been widely employed in various medical, dental, and biomedical applications.^10,11,52^ However, the balance between the efficacy and safety is crucial for any application. CuNPs have been associated with mitochondrial dysfunction, with loss of membrane potential and consequent activation of apoptotic pathways, mediated by increased ROS production and genomic lesions resulting from Fenton reactions.^53^ Studies indicate that, at low concentrations, CuNPs can positively modulate cellular antioxidant defenses; however, at high levels, they promote significant cytotoxicity.^54–56^ The exacerbated generation of ROS compromises vital biomolecular structures, such as DNA, lipids, and proteins, leading to programmed cell death. The structure and size of CuNPs modulate their biological interactions and the efficiency of ROS production.^55,57,58^ According to Cohen et al. (2013),^59^ the toxicity of CuNPs occurs by direct entry into the cell and subsequent intracellular release of Cu^2+^. Corroborating this, Sharma et al. (2021)^60^ observed that 11–14 nm CuNPs elicited oxidative stress and cytotoxicity in RAW 246.7 macrophages, as demonstrated by cell viability assays. Such analysis may also explain the cytotoxicity of copper oxide­(I) led by its reactivity and Cu^2^ release.^60,61^

However, with a focus on studying the influence of light over surfaces with biocidal properties, where contact typically lasts only a few seconds (transient and nonrecurrent), these cytotoxicity values do not compromise the present work. The widely cited article by Borkow and Gabbay (2009)^56^ mentions that the risk of adverse reactions due to dermal contact with copper is considered extremely low, as copper is not a foreign element to the human body.

To conclude, a summary of the presented results indicates that achieving significant reductions in viral load depends not only on the virus being tested but also on factors such as the concentration of the active material, temperature, exposure time, and now, the illumination condition. Consequently, the present study’s approach, which uses relatively low-cost photoactive particles incorporated into an inert polymeric matrix by a simple deposition technique, holds significant promise for advancing research on photoactive materials and photofunctional surfaces to combat the spread of pathogens.

## Conclusions

Pure PVA films, as well as CuMPs/PVA, CuNPs/PVA, and Cu_2_OMPs/PVA films, obtained by a low-cost deposition technique, were tested in the dark and under visible light against a murine coronavirus of the same genus as SARS-CoV-2. The influence of light on the virucidal activity of the films was observed.

CuMPs/PVA films showed virucidal photoactivity against the enveloped virus after 30 min of contact only under white light illumination. Using the ICC-RT-qPCR assay to quantify this activity, it was found that the composite film reduced the viral load by 43.1% under white light illumination, compared to merely 6.8% in the dark, presenting a photoactive behavior.

The finding of the influence of illumination over the virucidal activity of copper-based films represents a promising step forward in the research of photoactive disinfectant surfaces, a globally relevant topic to combat the spread of pathogens, particularly on frequently touched surfaces, in public spaces, and in hospitals. The promise of these films lies in their ability to damage pathogen structures without relying on chemical agents and using solar energy as a power source once the copper band gap range enables visible-light-driven application.

## Supplementary Material



## Notes

Thermal cycling conditions used for RT-qPCR, primer and probe sequences used for MHV-3 detection by RT-qPCR, and RT-qPCR standard curve parameters followed by the cell culture and virus titration assay description (PDF)

## Abbreviations

AFM: atomic force microscopy
ATCC: American Type Culture Collection
CPE: cytopathic effect
DMSO: dimethyl sulfoxide
FBS: fetal bovine serum
GDOES: glow discharge optical emission spectroscopy
ICC-RT-qPCR: integrated cell culture and reverse transcription quantitative PCR
LED: light-emitting diode
MEM: minimum essential medium
MHV-3: murine hepatitis virus type 3
PBS: phosphate-buffered saline
PVA: poly­(vinyl alcohol)
ROS: reactive oxygen species
SARS-CoV-2: severe acute respiratory syndrome coronavirus 2
SEM: scanning electron microscopy
TCID_50_: 50% tissue culture infectious dose
