# Supplementary material for: Visible-Light-Driven Photoactivity of Copper/PVA Composite Films against Murine Coronavirus
Source: ACS Omega. 2025 Oct 1;10(40):46524–32. doi: 10.1021/acsomega.5c02542 (PMC12529175; doi:10.1021/acsomega.5c02542)
Supplement: Supplementary file 1 [file ao5c02542_si_001.pdf]

Supporting information

Visible-light-driven photoactivity of copper/PVA composite films against murine coronavirus

Aline L. Schio[1], Michele S. de Lima[1], Marina D. Giustina[2], Rafael D. Cadamuro[3], Catielen P. Pavi[3], Alexandre F. Michels[1], Gislaine Fongaro[3], Mariana Roesch-Ely[1,2] and Carlos A. Figueroa[1*]*

[1] Postgraduate Program in Materials Science and Engineering, University of Caxias do Sul, 95070-560 Rio Grande do Sul, Brazil.

[2] Biotechnology Institute, University of Caxias do Sul, 95070-560 Rio Grande do Sul, Brazil.

[3] Laboratory of Applied Virology, Department of Microbiology, Immunology, and Parasitology, Federal University of Santa Catarina, 88040-900 Santa Catarina, Brazil.

Corresponding authors

Aline Lucchesi Schio; email: alschio1@ucs.br

Carlos Alejandro Figueroa; email: cafiguer@ucs.br

RT-qPCR parameters

Table S1. Thermal cycling conditions used for RT-qPCR.

**Step**	**Temperature**	**Time**	**Cycles**
Reverse transcription	50 °C	20 min	1
Initial denaturation	95 °C	15 min	1
Denaturation (cycling)	95 °C	15 s	40
Annealing/Extension	60 °C	45 s	40

Table S2. Primer and probe sequences used for MHV-3 detection by RT-qPCR.

**Component**	**Sequence (5' → 3')**	**Type**	**Reference**
Probe (FAM)	FAM-ACATGCTACGGCTCGTGTAACCGAACTGT-MGB	Hydrolysis probe	Besselsen *et al.*, 2002[1]
Forward primer	GGAACTTCTCGTTGGGCATTATACT	Primer	
Reverse primer	ACCACAAGATTATCATTTTCACAACATA	Primer	

Table S3. RT-qPCR standard curve parameters.

Parameter	Value
Platform	RT-qPCR (TaqMan)
Standard concentrations (copies)	5.0E+03 – 5.0E+05
Ct range	26.97 – 33.30
Coefficient of determination	R^2 = 0.805
Amplification efficiency (%)	158.1%

Cell culture and virus titration. The L929 cell line (ATCC CCL1), stored in a cryogenic tube in liquid nitrogen containing 90% fetal bovine serum (FBS) and 10% dimethyl sulfoxide (DMSO), was thawed at room temperature and transferred to a sterile 75 cm^2 cell culture flask containing minimum essential medium (MEM, Gibco), supplemented with 10 % fetal bovine serum (FBS, Merck). The flask was placed in an incubator at 37 ± 2 °C with an atmosphere containing 5 % CO_2.

Mouse hepatitis virus (MHV-3), generously provided by Professor Dr. Clarice Weis Arns from UNICAMP, was used as a control in the experiments.[2]

The MHV-3 viral stock, also stored at -80 °C, was thawed at room temperature. To determine the viral titer, the 50 % tissue culture infectious dose ($TCID_{50}$) was calculated using the Reed-Muench method.[3]

REFERENCES

1. Besselsen DG, Wagner AM, Loganbill JK. Detection of rodent coronaviruses by use of fluorogenic reverse transcriptase-polymerase chain reaction analysis. Comp Med. 2002 Apr;52(2):111-6. PMID: 12022389.
2. Körner, R.W.; Majjouti, M.; Alcazar, M.A.A.; Mahabir, E. Of Mice and Men: The Coronavirus MHV and Mouse Models as a Translational Approach to Understand SARS-CoV-2. Viruses **2020**, 12, 880. https://doi.org/10.3390/v12080880
3. Reed, L. J.; Muench, H. A Simple Method of Estimating Fifty Percent Endpoints. Am. J. Hyg. 1938, 27 (3), 493–497. https://doi.org/10.1093/oxfordjournals.aje.a118408.
